# Intrathecal pain management with ziconotide: Time for consensus?

**DOI:** 10.1002/brb3.2055

**Published:** 2021-03-09

**Authors:** Georgios Matis, Pasquale De Negri, Denis Dupoiron, Rudolf Likar, Xander Zuidema, Dirk Rasche

**Affiliations:** ^1^ Department of Stereotactic and Functional Neurosurgery Faculty of Medicine and University Hospital Cologne University of Cologne Cologne Germany; ^2^ Department of Anaesthesia, Intensive Care and Pain Medicine San Giuliano Hospital Giugliano, Naples Italy; ^3^ Anesthesiology and Pain Department Institut de Cancérologie de l’Ouest ICO Paul Papin Angers France; ^4^ Department of Anaesthesiology and Intensive Care Klagenfurt Clinic Klagenfurt Austria; ^5^ Department of Anaesthesiology and Pain Medicine Diakonessenhuis Utrecht/Zeist Zeist The Netherlands; ^6^ Functional Neurosurgery and Neurosurgical Pain Therapy University Hospital Schleswig‐Holstein Lübeck Germany

**Keywords:** cancer pain, chronic pain, consensus, intrathecal therapy, pain management, ziconotide

## Abstract

This article summarizes recommendations made by six pain specialists who discussed the rationale for ziconotide intrathecal analgesia (ITA) and the requirement for evidence‐based guidance on its use, from a European perspective. Riemser Pharma GmbH (Greifswald, Germany), which holds the European marketing authorization for ziconotide, hosted the meeting. The group agreed that ITA is under‐used in Europe, adding that ziconotide ITA has potential to be a first‐line alternative to morphine; both are already first‐line options in the USA. Ziconotide ITA (initiated using a low‐dose, slow‐titration approach) is suitable for many patients with noncancer‐ or cancer‐related chronic refractory pain and no history of psychosis. Adopting ziconotide as first‐line ITA could reduce opioid usage in these patient populations. The group advocated a risk‐reduction strategy for all candidate patients, including compulsory prescreening for neuropsychosis, and requested US–European alignment of the licensed starting dose for ziconotide: the low‐and‐slow approach practiced in the USA has a better tolerability profile than the fixed high starting dose licensed in Europe. Of note, an update to the European Summary of Product Characteristics is anticipated in early 2021. The group acknowledged that the Polyanalgesic Consensus Conference (PACC) treatment algorithms for ziconotide ITA provide useful guidance, but recommendations tailored specifically for European settings are required. Before a consensus process can formally begin, the group called for additional European prospective studies to investigate ziconotide in low‐and‐slow dosing strategies, in different patient settings. Such data would enable European guidance to have the most appropriate evidence at its core.

## Graphical abstract

Adopting ziconotide as first‐line intrathecal analgesia in Europe could provide adequate pain relief and reduce the opioid burden in patients with chronic refractory pain and no history of psychosis. This paper provides preliminary recommendations for ziconotide usage in Europe, using evidence‐based risk‐reduction strategies, tailored for patients with cancer‐ or noncancer‐related chronic pain.

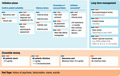

## 1 INTRODUCTION

Chronic pain remains a common and complex condition that is often challenging and burdensome at individual, clinical, and societal levels (Breivik et al., 2013). Choice of pharmacological agent and its route of administration are two of many components in a patient's individualized pain‐management strategy, but they are important aspects of care that have far‐reaching implications. Consequently, the development of new analgesics remains a focus of clinical research, a key aim of which is to reduce opioid use for severe and refractory pain (Jain et al., 2019). However, given the short‐term absence of novel, efficacious agents, it may be helpful to consider whether any licensed nonopioids could be better utilized; reflection on experience and evidence might identify practice adjustments that improve their clinical application. One such approach is intrathecal analgesia (ITA) with the nonopioid, ziconotide, which has been widely used in the USA for treating refractory cancer‐related and many forms of noncancer‐related pain since receiving US Food and Drug Administration (FDA) approval in 2004 (FDA, 2019). Ziconotide is little used in Europe despite being licensed by the European Medicines Agency (EMA) in 2005 (EMA SmPC, 2019). This article summarizes knowledge of ITA (and of ziconotide as a compound), explores differences between United States and European acceptance of ITA, and suggests proposals for initiating development of a European‐specific Consensus Statement on ziconotide use.

## 2 INTRATHECAL ANALGESIA: BACKGROUND AND PRINCIPLES

**FIGURE 1:**
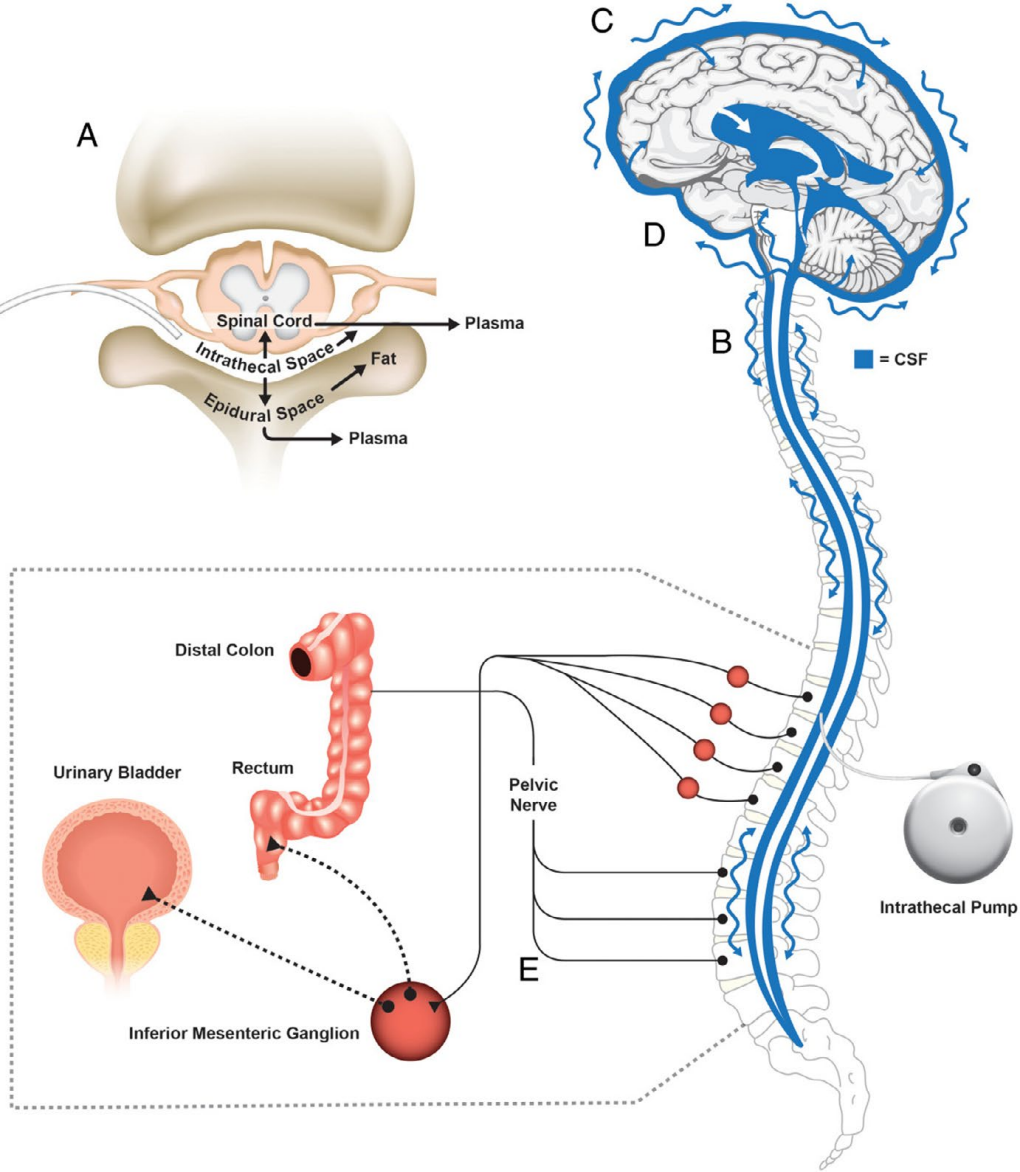
Intrathecal (IT) analgesia (ITA): central and peripheral sites related to the mechanisms of action of morphine and ziconotide ITA formulations, and common adverse effects. (a) Intrathecally administered drugs spread out of the IT space through the spinal cord into the epidural space, then enter systemic circulation, which might produce systemic adverse events. (b) ITA travels with the pulsatile motion of the cerebrospinal fluid (CSF) into the brain. (c) At high concentrations, ziconotide ITA might enter cortical regions, possibly leading to the development of neuropsychiatric events (e.g., cognitive impairment, psychosis). (d) Because morphine and ziconotide ITA travel with the pulsatile motion of the CSF, brainstem activity may be affected, causing systemic events (e.g., nausea, somnolence, headache); morphine ITA can also suppress respiratory centers in the brainstem, causing respiratory depression. (e) Morphine ITA spreads from the IT space into gastrointestinal and urinary systems and can act on opioid receptors involved with voiding urine and feces, leading to urinary retention and constipation. Reproduced from (Webster, 2015) with permission from Wiley Periodicals Inc

Intrathecal analgesia is licensed in morphine‐ or ziconotide‐based monotherapy regimens and is effective for many forms of chronic cancer‐ or noncancer‐related pain of neuropathic or nociceptive etiology (Deer, Hayek, Pope, et al., 2017; Deer, Pope, Hayek, Bux, et al., 2017; Deer, Pope, Hayek, Lamer, Veizi, et al., 2017; Deer et al., 2019; Hayek & Hanes, 2014). Emerging data also indicate that multidrug ITA regimens can effectively treat refractory cancer‐related pain (Caravajal et al., 2018; Dupoiron, 2019; EMA SmPC, 2019). ITA offers specific advantages for patients intolerant of (or refractory to) oral or systemic analgesia. For example, by delivering the agent directly into the cerebrospinal fluid, first‐pass metabolism, and the blood–brain barrier are bypassed (Smith & Deer, 2009). ITA therefore facilitates pain reduction using much smaller doses of active compound than are required with other administration routes (Smith & Deer, 2009; Webster, 2015) (Figure 1). In addition, compared with systemic or oral analgesia, ITA is associated with a lower incidence of long‐term systemic adverse effects, such as nausea or constipation (Hayek & Hanes, 2014; Pope & Deer, 2015a, 2015b; Webster, 2015).

In the USA, pain‐management algorithms for ITA, developed by the Polyanalgesic Consensus Conference (PACC) have guided its clinical application (Deer et al., 2007; Deer et al., 2012; Deer, Pope, Hayek, Lamer, Veizi, et al., 2017). Consequently, ITA, in general (with ziconotide in particular), is widely used (McDowell & Pope, 2016). However, in Europe, ITA is practiced in few specialist centers, and most regimens involve morphine rather than ziconotide, although where ziconotide is used the experiences are positive (McDowell et al., 2020). The low adoption of ziconotide ITA in Europe can be traced to key, inter‐related differences between the United States and European practices, in particular:

- European concerns regarding ziconotide dose selection and tolerability (high starting dose; poor tolerability): these concerns relate to the ziconotide dosing regimen listed in the EU Summary of Product Characteristics (SmPC) (Table 1)
- Limited recent European‐specific clinical data and formal guidance on ziconotide ITA (Alicino et al., 2012; Dupoiron et al., 2012; Dupoiron et al., 2019; Raffaeli et al., 2011): this clinical literature does not align with the dosing regimen in the EU SmPC

**TABLE 1:** Comparison of current US Food & Drug Administration (FDA) and European Medicines Agency (EMA) Summary of Product Characteristics (SmPC), Polyanalgesic Consensus Conference (PACC) treatment algorithms, and other recommendations for ziconotide administration; closer alignment of the EMA SmPC with the FDA SmPC is anticipated in early 2021

Parameter	FDA SmPC	EMA SmPC	Other recommendations
Maximum daily dose	19.2 µg/day (*0.8* µg/h)	21.6 µg/day	19.2 µg/day (*0.8* µg/h) ^a^
Starting dose	≤2.4 µg/day (*0.1* µg/h)	2.4 µg/day	0.5–1.2 µg/day (*0.02–0.05* µg/h)^a^; initiation with ≤ 0.5 µg/day (0.*02 *µg/h) may be preferred^b^
Dose increments	≤2.4 µg/day (*0.1* µg/h)	≤2.4 µg/day	≤0.5 µg/day (≤*0.02* µg/h) on a no more than weekly basis^b^, according to individual patient's pain reduction and tolerability (Fisher; Prager^b^)
Minimum interval between dose increases	≤2–3/week (*56–84* h)	24 hr	Titration slow and not more than once weekly^b^
Recommended interval (safety)	≤2.4 µg/day and ≤2–3/week	≥48 hr	Not more than once weekly^b^
Minimum concentration, external pump reservoir	5 µg/ml; change dose rate by adjusting flow rate or solution concentration	5 µg/ml	–
Minimum concentration, internal pump reservoir	25 µg/ml	25 µg/ml	–

Note

**Sources:** (FDA SmPC, 2019); (EMA SmPC, 2019).

^a^ (Deer, Hayek, Pope, et al., 2017; Deer, Pope, Hayek, Bux, et al., 2017; Deer, Pope, Hayek, Lamer, Veizi, et al., 2017).

^b^ (Fisher et al., 2005; McDowell & Pope, 2016; Prager et al., 2014).

Concerns regarding the high starting dose and consequent poor tolerability of ziconotide do not apply in the USA because since ziconotide became available, much work has been undertaken by PACC to refine its dosing and administration regimens. Consequently, the PACC pain‐management algorithms particularly suit the needs of US healthcare providers (Deer et al., 2007; Deer et al., 2012; Deer, Hayek, Pope, et al., 2017; Deer, Pope, Hayek, Bux, et al., 2017; Deer, Pope, Hayek, Lamer, Veizi, et al., 2017). For example, ziconotide dose and titration recommendations in these algorithms are lower, slower, and more detailed than information included in either the FDA or EMA SmPC literature (Deer, Hayek, Pope, et al., 2017; EMA SmPC, 2019; FDA, 2019; McDowell & Pope, 2016) (Table 1); of note, an update to the EMA SmPC, which will more closely align the European with the US dosing information is anticipated in early 2021.

The PACC algorithms therefore provide useful guidance on ziconotide administration but cannot be applied in European settings without considerable adaptation, given the differences between guidance and labeling information. The issue is not merely about dosing, however. Historically, there have also been differences in the provision of pain‐management services between Europe and the United States: In Europe, chronic pain management—including opioid prescribing—is only now becoming a clinical specialty (unlike in the United States, where it is a well established branch of medicine). Instead, pain control in Europe has often been managed by primary‐care or other nonspecialist physicians (O’Brien et al., 2017), with very few specialist pain clinics.

Local levels of experience with ITA may be limited in European health settings, not only because of the lack of specialist pain clinics but also because of reluctance to administer ITA, particularly for noncancer‐related pain, other than spasticity. This reluctance has stemmed from lack of compelling data on efficacy, safety, and cost‐effectiveness of ITA systems for noncancer pain, or evidence relating to nonopioid ITA use across diverse patient populations (Bottros & Christo, 2014; Deer et al., 2019).

Compared with the USA, European health settings have generally had less reliance on (or patient expectation of) high‐strength pharmacological interventions, including opioid analgesia for treating chronic noncancer‐related pain (DeWeerdt, 2019). With a lower opioid burden comes a lower requirement for opioid‐sparing alternatives, although there are signs that opioid‐related deaths are rising in many European countries, and it remains possible that an opioid epidemic could develop, in future, outside the United States (DeWeerdt, 2019).

Before European guidelines on ziconotide can be developed, it is therefore important to undertake local research and discussion, to fully explore the pharmacologic attributes and practical application of ziconotide across Europe; it is also important to understand, and then overcome, the barriers that have previously limited the use of ITA, in general, and specifically with ziconotide. Certainly, research is being undertaken in European centers with experience of ziconotide: early findings indicate the efficacy, safety, and tolerability of low‐and‐slow dose regimens in patients with chronic cancer‐ or noncancer‐related pain (Brinzeu et al., 2019; Caravajal et al., 2018; Matis & Visser‐Vandewalle, 2019). Indeed, guidelines on the application of ziconotide ITA in French palliative‐care settings have recently been published (Haute Autorité de Santé, 2020). However, no published recommendations consider ITA from a pan‐European clinical perspective, or feature a wide range of patient types, therefore, much additional work is needed.

In summary, until recently, lack of specialist pain‐management services across European health settings, lack of requirement for opioid‐sparing drugs (due to lack of any noteworthy opioid drug burden), and lack of consistency between the US clinical guidance on low‐dose titration and EU product licensing information for ziconotide have limited awareness and use of nonopioid ITA outside the USA. Changes in clinical access to pain‐management services, increasing evidence of a global opioid epidemic and alignment of US and EU SmPC information on low‐dose initiation of ziconotide ITA mean it is now necessary to re‐evaluate this agent as a viable first‐line alternative to opioid analgesia for chronic cancer‐ and noncancer‐related pain control.

## 3 ZICONOTIDE: PHARMACOLOGICAL SUMMARY

Before discussing clinical aspects, it is helpful to summarize the attributes of ziconotide that make it an attractive candidate for chronic, refractory pain management. Ziconotide is a synthetic, water‐soluble cone snail venom‐derived peptide with a molecular weight of 2,639 Daltons. In the systemic circulation, it is rapidly degraded by Phase I hydrolytic enzymes that are ubiquitous in the body, but it does not interact with cytochrome P450 enzymes (Pope & Deer, 2013). Ziconotide does not easily cross the blood‐brain barrier, instead revealing its highly potent antinociceptive effect only after intrathecal administration (Smith & Deer, 2009).

**FIGURE 2:**
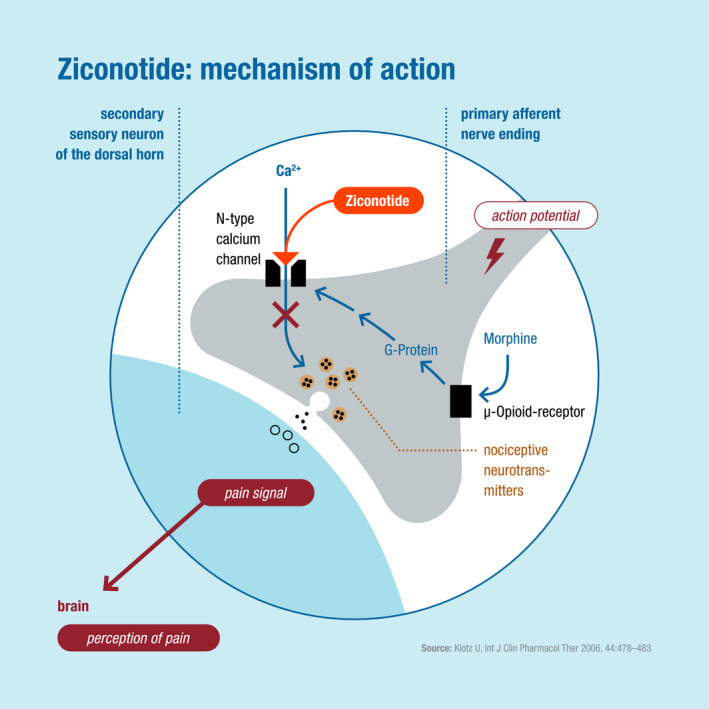
Mechanisms of action of ziconotide, a nonopioid analgesic administered intrathecally for chronic, refractory cancer‐ or noncancer‐related pain (modified from Klotz, 2006, with permission)

Ziconotide is a nonopioid analgesic that selectively binds to N‐type voltage‐sensitive calcium channels on primary nociceptive afferent nerves in the dorsal horn of the spinal cord (Deer et al., 2019). This mechanism releases analgesic neurotransmitters into the synaptic gap and subsequently blocks pain signal transmission (Figure 2) (Klotz, 2006; Pope et al., 2017). Because it has a narrow therapeutic window, careful dose titration, and a lag time to allow for onset (and offset) of analgesia and adverse effects are required (Deer, Hayek, Pope, et al., 2017; Deer, Pope, Hayek, Bux, et al., 2017; Deer, Pope, Hayek, Lamer, Veizi, et al., 2017; Pope et al., 2017; Schmidtko et al., 2010). These aspects—in part—influenced the low‐and‐slow dose and titration strategies developed by PACC (Deer, Hayek, Pope, et al., 2017; Deer, Pope, Hayek, Lamer, Veizi, et al., 2017), but given that they do not concur with the current EMA SmPC, may have driven the cautious approach and slow uptake of ziconotide across Europe.

It is important to consider why the EMA SmPC includes such a high, fixed starting dose for ziconotide ITA. The answer lies in the pivotal clinical studies of ziconotide, which used aggressive titration schedules involving an initiation dose of 2.4 μg/day (Rauck et al., 2006; Staats et al., 2004; Wallace, 2006). Ziconotide was licensed on the basis of these data because of the good efficacy and overall safety findings reported, although the high‐fixed initiation dose was associated with a high rate of adverse effects including neuropsychotic episodes, confusion, and nausea (Rauck et al., 2006; Staats et al., 2004; Wallace, 2006). With the benefit of hindsight, it is therefore understandable why European clinicians have been reluctant to use ziconotide as a first‐line ITA, if they align their practice with the current EMA SmPC.

### 3.1 Stability and degradation of ziconotide

Although the stability of ziconotide can be positively influenced by coadministration with morphine (Dupoiron et al., 2014)—therefore, a combination regimen has pharmacological merit) (Carvajal et al., 2018; Dupoiron, 2019; Dupoiron et al., 2020)—multidrug regimens represent off‐label usage and are not permitted by ITA pump manufacturers. In addition, the PACC guidelines note that there is no evidence basis to advocate coadministration of ziconotide and opioid ITA, or confirm the stability of admixtures in multidrug ITA regimens (Deer, Pope, Hayek, Bux, et al., 2017; Deer, Pope, Hayek, Lamer, Veizi, et al., 2017).

It is important to note that temperature (including presence of fever in the patient), altitude (air travel or living at high‐altitude), and light exposure (during transportation or storage) affect the stability of ziconotide (Bazin et al., 2015; EMA SmPC, 2019).

Implanted pumps must be refilled every 3–4 weeks when ziconotide is diluted with preservative‐free sodium chloride 9 mg/ml (0.9%) solution for injection. Dilution is essential during the trialing phase, but degradation of ziconotide takes place over time due to the decrease of L‐methionine in the pump (EMA SmPC, 2019).

## 4 EUROPEAN CONSENSUS STATEMENT ON ZICONOTIDE: PRELIMINARY CONSIDERATIONS

**FIGURE 3:**
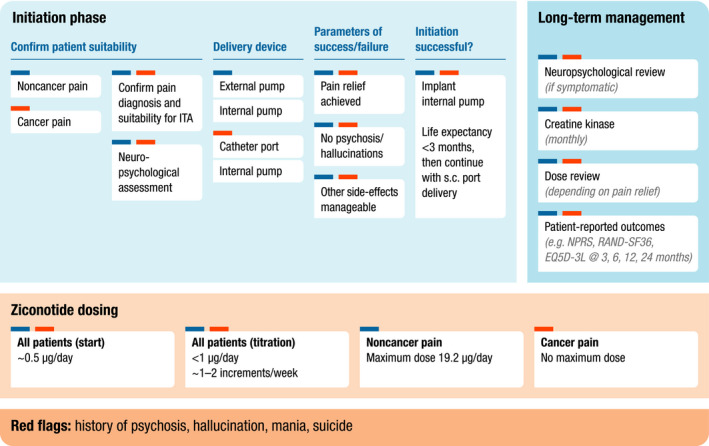
Infographic summarizing the key requirements for consideration in of any European Consensus Statement for initiation and long‐term management phases of ziconotide intrathecal analgesia (continuous infusion) (ITA). s.c., spinal catheter; NPRS, numeric pain rating scale; RAND‐SF36, Research and Development Corporation short‐form 36; EQ5D‐3L, EuroQol five‐dimension three‐level

Key requirements of any European Consensus Statement specific to ziconotide ITA will likely involve patient selection, drug‐trialing, pump choice, device implantation, ongoing administration, patient management, and outcomes assessment. This list is not limited; points listed below, and briefly illustrated in Figure 3, merely form the basis for future discussions.

### 4.1 Patient selection for ziconotide ITA

It is wise for European guidelines on ITA to reflect on PACC recommendations, which list ziconotide and morphine as first‐line options for active cancer‐ and noncancer‐related pain, regardless of whether pain is localized or diffuse (Deer, Pope, Hayek, Bux, et al., 2017; Deer et al., 2019). However, PACC guidelines emphasize that, unless contraindicated, ziconotide should be the first drug selected for noncancer patients (Deer, Pope, Hayek, Bux, et al., 2017): In contrast to opioids, it does not cause tolerance, dependence, or respiratory depression (Smith & Deer, 2009). PACC guidelines consider ziconotide to be particularly suitable for patients who cannot receive morphine (due to underlying conditions or opioid intolerance, for example) (Deer, Pope, Hayek, Bux, et al., 2017; McDowell & Pope, 2016).

The following points regarding patient selection should be considered from a European perspective, based on clinical evidence, experience, and local service provision:

- The position of ziconotide as first option for ITA (for cancer‐ and noncancer‐associated pain, in the era of the opioid epidemic, if there is no contraindication to its use
- The value of multidrug ITA regimens involving ziconotide, especially for refractory cancer pain
- The requirements for neuropsychiatric evaluation in all patients before and after commencing ziconotide ITA, regardless of pain etiology
- The confirmation of pain diagnosis and ITA suitability: ITA, in general, is not a panacea for all forms of refractory, severe chronic pain.

### 4.2 Rationale for Ziconotide as first‐line ITA

Morphine ITA was the gold standard for several years but ziconotide ITA has been far more widely investigated (reviewed in Bäckryd, 2018; Deer et al., 2019). With a greater depth of data comes a clear understanding of the potential position of this agent in the severe, refractory pain‐management paradigm.

Data from the Patient Registry of Intrathecal Ziconotide Management (PRIZM) study indicate that using ziconotide as first‐line ITA might offer better pain relief, and sustained efficacy, compared with using it subsequently (Deer et al., 2018). This small study identified improvements in numeric pain rating scale (NPRS) scores and pain relief (≥30% reduction from baseline NPRS score)—at 12 weeks and 18 months—in patients who received ziconotide ITA first, compared with those who received it as a subsequent ITA regimen (Deer et al., 2018). PRIZM used a low starting dose of ziconotide (1.2 μg/day, with an undiluted 25 μg/ml formulation); the safety profile observed in this study was consistent with that listed in the ziconotide prescribing information. Additional findings from PRIZM indicated that tolerance did not develop with ziconotide ITA (Deer et al., 2019; McDowell et al., 2020).

### 4.3 Neuropsychiatric evaluation

There can be little argument that all patients with noncancer‐related pain require thorough neuropsychiatric assessment before commencing ziconotide ITA (Deer, Hayek, Pope, et al., 2017; Deer, Pope, Hayek, Bux, et al., 2017). History of psychiatric illness (particularly psychosis, depression, or suicidal ideation) is an absolute contraindication to ziconotide (EMA SmPC, 2019). For cancer patients, however, PACC guidance suggests a more flexible approach (Deer, Hayek, Pope, et al., 2017; Deer, Pope, Hayek, Bux, et al., 2017), although pretrialing evaluation can be undertaken. Protocols for initial and ongoing neuropsychiatric monitoring in patients on ziconotide ITA require clarification from a European perspective, although further evaluation is only likely to be required if symptoms develop after implantation of the permanent pump.

### 4.4 Confirmation of pain diagnosis

Before starting ziconotide, the patient's pain diagnosis should be confirmed, to ensure that the pain is of nociceptive or neuropathic origin; ziconotide has been shown to be beneficial when other drugs have failed (Lux & Rasche, 2011). Although ziconotide ITA is effective for many types of cancer‐ and noncancer‐associated pain, it is not effective for global pain, headache, or facial pain and should not be trialed in patients presenting with these symptoms (EMA SmPC, 2019).

### 4.5 Trialing strategies: overview

Trialing is widely considered necessary to test a patient's efficacy response to ziconotide while limiting the risk of adverse effects (McDowell & Pope, 2016). Consequently, trialing has multiple aims, of equal importance in clinical practice. Low‐and‐slow dose titration, adapted for each patient, rather than rushing toward achieving clinically meaningful pain relief, is the objective (reviewed in McDowell & Pope, 2016) (Matis & Visser‐Vandewalle, 2019).

Potential side effects of ziconotide ITA including confusion and nausea are sometimes experienced during the trial phase, but psychosis is very rare when ziconotide is titrated in a low‐and‐slow approach. Any serious events that occur clearly impact on a decision to continue treatment (Deer, Hayek, Pope, et al., 2017; EMA SmPC, 2019). Of note, many patients receiving ziconotide ITA do not experience complications during the trial period, but they can develop at any time during long‐term treatment (see the section, Adverse effects and contraindications).

Despite the need for individualization, the consensus process should establish frameworks for trialing such as administration method, starting dose, phasing of increments, and location and duration of the trial period, because the needs differ in specific patient subgroups. For example:

- Full trialing is widely considered necessary for first‐line ITA in patients with noncancer‐related pain
- Although full trialing is unnecessary for patients with cancer‐related pain, initial dosing should be titrated, to reduce risk of complications

### 4.6 Ziconotide administration during the trial

There are three main approaches to trialing ziconotide, all of which are undertaken in in‐patient settings, to monitor safety and efficacy responses:

- Bolus administration (using syringe devices rather than pumps):
- Flexible‐dose administration (e.g., bolus night‐time dosing in addition to pump administration)
- Initiation with low‐dose, slow‐titration therapy (continuous intrathecal infusion using internal or external pumps).

Although bolus dosing is included in PACC algorithms and widely described in the literature (Bäckryd et al., 2015; Deer, Hayek, Pope, et al., 2017; Deer, Pope, Hayek, Bux, et al., 2017; Mohammed et al., 2013), there are concerns about advocating this approach in any European consensus statement. Bolus dosing requires higher quantities of drug than pump administration; given the hydrophilic nature of the ziconotide molecule, this approach is likely to result in inconsistencies in manual administration, slow tissue penetration, and variability in how the dose circulates within cerebrospinal fluid (reviewed in McDowell & Pope, 2016).

Nevertheless, the efficacy of a flexible‐dose ziconotide regimen at initiation was demonstrated using a bolus‐and‐implantation regimen (dose range 1–4 μg per day (Pope & Deer, 2015b). A conservative low‐dose approach with pump implantation, albeit after an initial bolus trial, was successful in 15 patients with chronic noncancer pain (Prusik et al., 2017). There is evidence that nocturnal dosing, in addition to low‐and‐slow titration, improves the initial tolerability of ziconotide (Deer et al., 2019; McDowell & Pope, 2016; Pope & Deer, 2015b).

Internal pumps are the optimum delivery systems for ITA administration and are recommended for patients with a life expectancy >3 months, once efficacy and safety of ziconotide have been demonstrated in the individual (Deer, Hayek, Pope, et al., 2017; Deer, Pope, Hayek, Bux, et al., 2017). Although ITA can be fitted in any patient before starting ziconotide, these devices are only advisable from the outset for people with cancer‐related pain. For those with noncancer‐related pain, surgical implantation of a “permanent” pump before trialing has a high cost and morbidity burden, at a stage when its therapeutic benefits are unknown. Trialing ziconotide in noncancer patients therefore usually requires temporary delivery methods to establish the initial efficacy and safety response.

Trialing ziconotide ITA using an external syringe pump is a widely used strategy (reviewed in Bäckryd, 2018), but there are concerns about this approach. Firstly, no external pump can reproduce the efficiency of an internal drug‐delivery system for ITA: syringe pumps exhibit different flow rates, which alter the pharmacodynamic parameters of ziconotide and therefore affect its efficacy and tolerability responses, as indicated for bolus dosing above. Also, external pumps suitable for ziconotide trialing are not available throughout Europe; any guidelines would need to take into account local access to devices.

A subcutaneous port, connected to an intrathecal catheter, is an option for patients with cancer‐associated pain who have a short life expectancy (<3 months). This procedure has a lower morbidity and cost burden than a permanently implanted pump (see section on trialing in patients with cancer‐related pain) (Dupoiron, 2019; Dupoiron et al., 2020; Haute Autorité de Santé, 2020).

### 4.7 Catheter tip placement

In addition to the choice of pump, catheter tip positioning is extremely important, and any European Consensus statement should include drug‐specific guidance on this aspect of administration. For ziconotide ITA, the catheter tip should be placed at the spinal level closest to the dermatomal region of pain. With lumbar pain, for example, the catheter tip should be at T8–T10 (Bäckryd, 2018; Deer, Pope, Hayek, Bux, et al., 2017; Deer, Pope, Hayek, Lamer, Veizi, et al., 2017; tip placement higher than T8 is associated with emergence of side effects including hallucinations. N‐Type voltage‐dependent calcium channels in the ventricle might be affected by ziconotide concentrations that do not cause such side effects when the catheter is placed at a lower position.

Requirements for catheter tip placement vary for other ITA agents and cannot be made without evaluating the pharmacodynamic profile and stability of the specific regimen concerned. CSF dynamics should be taken into account (Prager et al., 2014).

In all cases, catheter tip positioning must be an X‐ray guided procedure, with details of placement appropriately documented in the patient's notes.

### 4.8 Trialing ziconotide ITA in cancer‐related pain

The PACC algorithms state that full trialing of ziconotide ITA is unnecessary for patients with cancer‐related pain (although this has a low evidence level in PACC). This approach also applies in European settings, but risk of adverse events should be minimized by adopting one of the following strategies:

- Immediate surgical implantation of an internal pump in patients with life expectancy of >3 months, then commencing ziconotide ITA using a low‐and‐slow dose titration process
  ○ For patients with shorter life expectancy, a subcutaneous port should be used
- Administering ziconotide via intrathecal catheter port, to assess the patient's response prior to internal pump implantation
  ○ There is emerging evidence that ziconotide administration via intrathecal catheter may be an efficacious long‐term option for patients with cancer‐related pain (Dupoiron et al., 2020).

### 4.9 Trialing ziconotide in noncancer‐related pain

The PACC algorithms recommend that full trialing of first‐line ziconotide ITA is necessary for patients with noncancer‐related pain, to minimize the risk of complications (Deer, Hayek, Pope, et al., 2017; Deer, Pope, Hayek, Bux, et al., 2017; Deer, Pope, Hayek, Lamer, Veizi, et al., 2017); however, trialing is not universally performed in specialist pain centers. Discussion of the following points, from European perspectives, is required:

- How closely should titration follow the PACC recommendations rather than the FDA and EMA SmPCs for ziconotide?
- How long should the in‐patient phase typically take?
- What are the thresholds for therapeutic failure (e.g., emergence of specific adverse events; lack of meaningful pain response over time)?
- Should recommendations for combination regimens involving ziconotide ITA be developed? Monotherapy is the only licensed indication, yet there is emerging evidence for multidrug regimens, in cancer patients with short life expectancy or no other analgesic options

The European consensus process should explore each of these points from different clinical positions as there are very few clear answers at present. It is clear that the ziconotide trial phase should commence using an implanted or external (syringe) pump device; bolus administration (lumbar puncture injection) is not recommended. However, anticipated duration and parameters of ziconotide ITA trials in Europe require further discussion: requirements may differ locally, and recommendations given by PACC are likely to be specific to the US health sector (Deer, Pope, Hayek, Bux, et al., 2017). As a foundation for discussions, and in the absence of any local protocols, a trial period could be a minimum of ~1 week in‐patient stay (a maximum 4 weeks with an external pump), followed by implantation of the permanent pump, with the correct dose being found over the next few months, in outpatient consultations. Given different healthcare settings across Europe, it is likely that any recommendations on trial duration would have to include variability.

#### 4.9.1 Ziconotide dosing and tolerance

The low‐dose range for ziconotide initiation in the PACC algorithms (0.5–1.2 μg/day for cancer‐ and noncancer‐related pain; Table 1) (Deer, Pope, Hayek, Bux, et al., 2017) should form the basis of discussions in relation to the European Consensus guidelines for treatment initiation.

Certainly, the dose should be titrated upwards very slowly every 2–3 days until an efficacy response is observed. Although it is helpful to have a standard protocol, this acts as the foundation for individualized therapy: time taken to reach a target (efficacious) dose is not important. Broadly, in the patient with noncancer‐related pain, if no pain relief is observed when the total ziconotide dose is 10 μg/day and the patient has received ITA for 3 weeks, this would be a reasonable point to review the individual case as being a likely nonresponder. However, some patients may not experience a reduction in pain until higher doses are administered. Notably, the PACC guidelines include an upper threshold of 19.2 μg/day for achieving the initial response. Before classifying a case as a treatment failure, it is important to establish whether any technical or human factors may have resulted in the lack of response. If nothing can be identified, ziconotide ITA should be stopped.

There is no maximum daily dose of ziconotide for patients with cancer‐related pain, if the patient tolerates ziconotide and it continues to provide pain relief. There is no evidence that patients with chronic pain conditions develop tolerance to ziconotide even with long‐term use (Smith & Deer, 2009). On the contrary, dosages can often be reduced over time, with efficacy maintained (Deer et al., 2018; Deer et al., 2019; Schmidtko et al., 2010; Webster, 2015).

#### 4.9.2 Adverse effects and contraindications

Adverse effects can emerge in patients on ziconotide ITA at any point from initial dose titration through to after several years of successful treatment (EMA SmPC, 2019). Ziconotide ITA should stop immediately if psychosis or hallucinations occur (EMA SmPC, 2019), but other adverse events can be managed on an individual basis. More research and discussion is required, to evaluate strategies that alleviate complications but avoid complete discontinuation. For example, halving the ziconotide dose and increasing the frequency of patient monitoring (with gradual up‐titration, if the adverse event remains controlled) may provide ongoing pain management, without having to initiate a new analgesic.

The most common cognitive and neuropsychiatric symptoms reported with ziconotide ITA typically occur after several weeks’ treatment and include confusion, hallucinations, paranoia, aggression, delirium, mania, or psychosis. Severe, refractory pain is associated with a heightened risk of suicidal ideation, and ziconotide may exacerbate depression or suicide risk in susceptible patients. However, it is important to evaluate whether the events are a consequence of factors other than ziconotide use (e.g., concomitant medications or HRQoL issues associated with underlying disease). Cognitive effects associated with ziconotide usually abate within 4 weeks after treatment stops, but may persist in some cases.

Elevations in creatine kinase were reported in some patients receiving ziconotide in clinical trial settings (Rauck et al., 2006; Staats et al., 2004; Wallace, 2006). These elevations were usually asymptomatic and rarely progressed. Although the PACC guidelines, FDA and EMA SmPCs recommend that creatine kinase levels are monitored at baseline and regularly in all patients on ziconotide ITA, such guidance lacks clarity (EMA SmPC, 2019; FDA, 2019). There is anecdotal evidence that creatine kinase levels are only monitored when there is clinical suspicion. The European SmPC states that ziconotide could be discontinued if progressive or clinically significant elevations in creatine kinase and clinical features of myopathy or rhabdomyolysis, emerge. Again, this is a topic for further investigation from a European standpoint.

Ziconotide is contraindicated in combination with intrathecal chemotherapy. Few patients have received systemic chemotherapy and IT ziconotide concomitantly, therefore, although such treatment is not contraindicated, caution should be exercised, with careful monitoring.

Ziconotide ITA should be discontinued if a patient has depressed levels of consciousness; in such situations, the patient typically remains conscious and breathing is normal. Withdrawal of concomitant medications that are known to be CNS depressants should also be considered. Increased somnolence has been noted with concomitant ziconotide ITA and systemic baclofen, clonidine, bupivacaine, or propofol, and their simultaneous use is discouraged (Smith & Deer, 2009).

Patients receiving ITA, their careers and physicians must be vigilant for typical signs of meningitis, given the increased risk of infections in patients with catheters or implanted pumps.

#### 4.9.3 Efficacy outcomes assessment

Comprehensive outcome assessment in patients receiving ITA was not addressed in the PACC guidelines; it remains an unmet research and clinical need. Before European consensus guidelines can be discussed, it is necessary to consider what constitutes a positive outcome, and which instruments are valid in clinical practice. Pain scales including the Visual Analogue Scale (VAS) or NPRS are widely used in research, and a systematic meta‐analysis of Phase 3 studies of ziconotide (which used data obtained using these instruments) has indicated that treatment led to beneficial reductions in chronic pain (Brookes et al., 2017).

However, many patients for whom ITA is indicated have complex needs, and reaching the research benchmark (namely, of a pain reduction threshold of ≥30% compared with baseline) may be unattainable in real‐world settings. When few options are available to an individual, even a minor reduction (e.g., a 10% improvement in pain relief, or a less‐disturbed night's sleep) might be a satisfactory outcome. Conversely, lowering a pain reduction threshold to <30% might mean that a placebo effect would positively—and incorrectly—influence the outcome. Instruments for evaluating patient response that might be better suited for clinical use include the Global Disability Scale or EuroQoL 5‐dimension questionnaires, which measure a broader range of parameters than the VAS or NPRS.

However, caution is also required in terms of the dependent relationship between reimbursement of treatment costs and “successful outcome.” Success can be defined by different metrics, given the diverse range of health‐insurance models across Europe. Any European Consensus document should therefore include open wording that encompasses pain scales and appropriate questionnaires in relation to outcomes assessments. Such wording could be “the aim is a pain reduction of at least 30% on the VAS scale *OR* a marked improvement in the parameters of accepted questionnaires.” Indeed, it may be advisable that recommendations for outcomes assessment are made by an independent third party, such as an interdisciplinary working group of pain experts, rather than falling solely within the remit of any Consensus group.

## 5 AREAS FOR ADDITIONAL FOCUS

Ziconotide ITA has the potential to be a cost‐effective pain‐management strategy for many forms of chronic, severe cancer‐ or noncancer‐related pain (Deer et al., 2019]. Of utmost importance is that this therapy is administered by physicians who are comfortable initiating and managing ITA, who are supported by a healthcare infrastructure that provides all aspects relevant to ITA (implantation, maintenance, programming, reservoir refills, and troubleshooting).

Before consensus guidelines for ziconotide ITA are drafted, further research and peer discussion is required, including (but not limited to) the following points, many of which are beyond the scope of the present paper:

- Safety, efficacy, and clinical use of ziconotide in admixtures and multidrug ITA regimens
- Switch protocols from opioid ITA to ziconotide ITA (addressing opioid dose reduction during the switch, and any opioid withdrawal effects)
- Evaluation of low‐dose titration strategies for ziconotide ITA in different European settings
- Evaluation of optimum catheter placement strategies
- Additional cost‐benefit analyses of ziconotide ITA, including patient‐reported and HRQoL outcomes beyond pain‐scale measurements

## 6 CONCLUSIONS

This initial consensus statement is based on clinical experiences and views of pain‐management specialists who participated in the roundtable discussion, together with further consideration of published literature relating to ziconotide. Ultimately, the group recommends that a European‐specific consensus document is created, to guide clinical use of ziconotide ITA. Ziconotide ITA (both as monotherapy and in multidrug regimens) is an under‐utilized agent that offers great potential in Europe for treating many forms of severe, refractory, cancer‐, or noncancer‐related pain. Any consensus document should reflect on the PACC pain‐management algorithms for ziconotide ITA (which were created for the US health sector), and recent palliative care recommendations in France; in both existing guidelines, ziconotide is a first‐line ITA, not a last‐resort for pain relief.

Pan‐European guidelines would need to allow flexibility, to suit different healthcare models across the continent. Before any guidelines can be prepared, further research investigating dosing and administration of ziconotide ITA in European patients with cancer‐ or noncancer‐associated chronic pain is required. Research priorities include evaluation of low‐and‐slow dose titration protocols, internal and external pumps (and catheter ports) for trialing ITA, multidrug ITA regimens, and switch protocols with opioid‐based ITA. If ziconotide ITA is shown to be a practical, efficacious and well‐tolerated option, research and discussion should also consider its position in the pain‐management pathway (to ensure that patients who might benefit most from ziconotide receive it at the appropriate time for their health and well‐being).

Pre‐existing concerns relating to ziconotide use in Europe must be properly investigated through sharing clinical experience, and evaluating data, during the consensus process. There is already good evidence from the USA that, following appropriate screening and low‐and‐slow dosing protocols, ziconotide ITA is an effective alternative to morphine‐based ITA with good long‐term safety and tolerability. If similar strategies can be devised and adopted in Europe, wider implementation of ziconotide ITA might follow. Ultimately, this could help to fulfill unmet needs for management of chronic, refractory pain by extending analgesic choice in this time of the global opioid epidemic.

## CONFLICTS OF INTEREST

Dr Matis received consultant fees from Boston Scientific and Riemser Pharma GmbH. Dr De Negri received a consultant fee from Riemser Pharma GmbH. Dr Dupoiron received consultant fees from Medtronic and Riemser Pharma GmbH. Dr Likar received consultant fees from Grünenthal, Sanofi, MSD, Mundipharma, Pfizer and Riemser Pharma GmbH. Dr Zuidema received a consultant fee from Riemser Pharma GmbH. Dr Rasche received consultant fees from Abbott, Boston Scientific, Medtronic and Riemser Pharma GmbH.

## AUTHOR CONTRIBUTIONS

All authors participated actively in the meeting and contributed to the development of the consensus manuscript, including approval of the final draft.

### PEER REVIEW

The peer review history for this article is available at https://publons.com/publon/10.1002/brb3.2055.
